# Narrative impairment, white matter damage and CSF biomarkers in the Alzheimer’s disease spectrum

**DOI:** 10.18632/aging.102391

**Published:** 2019-10-31

**Authors:** Claudia Drummond, Gabriel Coutinho, Marina Carneiro Monteiro, Naima Assuncao, Alina Teldeschi, Andrea Silveira de Souza, Natalia Oliveira, Ivanei Bramati, Felipe Kenji Sudo, Bart Vanderboght, Carlos Otavio Brandao, Rochele Paz Fonseca, Ricardo de Oliveira-Souza, Jorge Moll, Paulo Mattos, Fernanda Tovar-Moll

**Affiliations:** 1 Department of Neuroscience, D’Or Institute for Research and Education (IDOR), Rio de Janeiro, Brazil; 2 Department of Speech and Hearing Pathology, Federal University of Rio de Janeiro, Rio de Janeiro, Brazil; 3 Graduate Program in Morphological Sciences, Institute of Biomedical Sciences, Federal University of Rio de Janeiro, Rio de Janeiro, Brazil; 4 Department of Psychology, Celso Lisboa University Center, Rio de Janeiro, Brazil; 5 Neurolife – Cerebrospinal Fluid Specialized Laboratory, Rio de Janeiro, Brazil; 6 Laboratory of Clinical and Experimental Neuropsychology, Department of Psychology, Pontificial Catholic University of Rio Grande do Sul, Porto Alegre, Brazil; 7 Department of Psychiatry and Forensic Medicine, Institute of Psychiatry, Federal University of Rio de Janeiro, Rio de Janeiro, Brazil

**Keywords:** language, white matter, mild cognitive impairment, Alzheimer’s disease, CSF biomarkers

## Abstract

Background: Narrative discourse (ND) refers to one’s ability to verbally reproduce a sequence of temporally and logically-linked events. Impairments in ND may occur in subjects with Amnestic Mild Cognitive Impairment (aMCI) and Alzheimer’s Disease (AD), but correlates across this function, neuroimaging and cerebrospinal fluid (CSF) AD biomarkers remain understudied.

Objectives: We sought to measure correlates among ND, Diffusion Tensor Imaging (DTI) indexes and AD CSF biomarkers in patients within the AD spectrum.

Results: Groups differed in narrative production (NProd) and comprehension. aMCI and AD presented poorer inference abilities than controls. AD subjects were more impaired than controls and aMCI regarding WB (p<0.01). ROIs DTI assessment distinguished the three groups. Mean Diffusivity (MD) in the uncinate, bilateral parahippocampal cingulate and left inferior occipitofrontal fasciculi negatively correlated with NProd. Changes in specific tracts correlated with T-tau/Aβ1-42 ratio in CSF.

Conclusions: AD and aMCI patients presented more ND impairments than controls. Those findings were associated with changes in ventral language-associated and in the inferior parahippocampal pathways. The latest were correlated with biomarkers’ levels in the CSF.

Methods: AD (N=14), aMCI (N=31) and Control (N=39) groups were compared for whole brain (WB) and regions of interest (ROI) DTI parameters, ND and AD CSF biomarkers.

## INTRODUCTION

Ancillary methods for assessing cognitive impairment within the *continuum* of Alzheimer's Disease (AD) have enabled substantial progress in the understanding of the pathophysiology of the disease, which may ultimately contribute to the development of strategies for prevention, early diagnosis and disease-modifying treatment [1]. Measurable indicators of AD pathology comprise an array of clinical, biochemical and neuroimaging factors, such as: (1) changes in structural MRI (hippocampal and entorhinal cortex atrophy, for example); (2) abnormalities in the white matter (WM) integrity evidenced by Diffusion Tensor Imaging (DTI) [2]; (3) regional glucose metabolic reduction, as measured by 2-[18F]-fluoro-2-deoxy-d-glucose PET (FDG-PET) [3]; (4) cortical amyloid load demonstrated through the Carbon-11-labelled Pittsburgh compound B (11 C-PiB) PET [3]; (5) molecular alterations of the brain (metabolomics, oxidative stress, beta-amyloid processing, tau-protein pathology and insulin signaling) [4–6] and (6) cognitive deficits indicated by neuropsychological assessment [7–9]. In this respect, recent evidence suggested that combining results from different categories of AD markers may improve the accuracy for both the detection of dementia and for the prediction of cognitive decline in subjects with Mild Cognitive Impairment (MCI) [10–14]. However, experience in multimodal analyses is incipient and recommendations for the most useful composite data are still not available.

Challenges for progressing research in this field include some uncertainties at each measurement level. Although MCI subjects with prominent memory impairments (amnestic MCI - aMCI) may present higher risk for developing dementia than the non-amnestic subtype [15], it has become increasingly accepted that early deficits in other cognitive domains, such as language, could be observed in those cases [16–18]. Yet, research on the linguistic aspects of MCI (and early AD) has been narrowed down to a limited number of its components. For example, in addition to difficulties in visual naming [19] and lexical retrieval by semantic criteria tasks [20], evidence from the literature indicated that subjects with AD may show early impairments in narrative discourse [16, 21–25]. Narrative Discourse is a complex linguistic function, which refers to one’s ability to logically, temporally and casually-integrate data. Its assessment usually requires verbal reproduction of sequences of logically-interconnected events within a specific scenario and characters. This high-order linguistic ability requires the conjugation of different components of language (phonological, lexical, semantic, morphosyntactic and pragmatic) with diverse cognitive functions and social demands - such as memory, planning, the ability to create mental models and draw inferences from different contexts and interlocutors [26]. ND is essential for efficient communication capacity and the detection of changes in those abilities may be early indicators of aging-related cognitive decline in clinical practice.

When it comes to identifying MCI, it has been suggested that those subjects may not be discernible from normal aging using basic screening tasks [27]. Hence, assessment of complex cognitive abilities, which rely on processing and integrating data from different cognitive functions, appears to be of greater value for the detection of those cases [28].

Moreover, knowledge of neural networks underlying narrative discourse has been concentrated on its comprehensive component (Narrative Comprehension -NComp), whereas investigations on Narrative Production (NProd) have not advanced at the same rate for functional magnetic resonance imaging (fMRI) studies [29, 30]. On the other hand, data from researches using Diffusion Tensor Imaging (DTI) have revealed abnormalities in the uncinate fasciculus in MCI patients compared to controls, which were associated with high risk for progression to dementia [31]. Specific pathways have been implicated in language impairments - the superior longitudinal fasciculus (SLF), the inferior longitudinal fasciculus (ILF), the inferior fronto-occipital fasciculus (IFOF), the corpus callosum (CC), and the uncinate fasciculus (UNC) [32–39], nevertheless evidence for the associations between clinically observed language impairments and WM integrity remains scarce in the literature. Furthermore, data on the relationships among neuropsychological and neuroimaging findings in the AD spectrum and cerebrospinal fluid (CSF) biomarkers (beta-Amyloid 1-42 – Aβ1-42 - and tau protein) of AD pathology are inconclusive across studies [40]. Determining correlations between DTI and CSF biomarkers could shed light into whether WM compromise and neurodegeneration are independent or associated features in AD physiopathology [41].

The rationale for assessing subjects with MCI and AD through a multimodal approach resides on the assumption that combining neuropsychological, neuroimaging and biochemical elements could provide a comprehensive appreciation of the underlying basis of the cognitive impairments in the early AD-spectrum. Thus, the present study aimed to investigate the correlates among narrative discourse deficits in aMCI and AD patients, the WM circuitry integrity and the levels of AD-related CSF biomarkers. We hypothesized that deficits in narrative discourse could be related to anatomical damage to the fasciculi most directly engaged in language. We also predicted that lower WM integrity in those tracts could be associated with a higher total-tau (t-tau)/Aβ1-42 ratio, reflecting correspondent neuronal injury due to AD pathology.

## RESULTS

No significant differences were identified across groups regarding sex, age and education (Table 1). As expected, mean scores on the MMSE were significantly lower for AD subjects compared to controls and aMCI groups; no differences were found for this variable between controls and aMCI. Groups did not differ on GDS scores (*p* = 0.345). All groups performed significantly different in the Boston Naming Test, the Digit Span backward and the RAVLT (Ratio A7/A5). Participants with aMCI scored significantly higher in semantic VF compared to AD subjects; in phonemic VF mean scores were lower for aMCI than for controls. Table 2 depicts data from cognitive and behavioral assessment.

**Table 1:** Demographic comparisons of controls and patients.

	**Control**		**aMCI**		**AD**			
	**Mean**	**SD**	**Mean**	**SD**	**Mean**	**SD**	**F**	**P**
N (total)	39		31	14		
Age	**71.77**	4.3	**72.16**	4.8	**75.29**	6.8	2.70	0.073
Years of education	**14.26**	2.3	**13.16**	2.5	**13.07**	2.9	2.15	0.123
Sex (F/M)	24 / 15		16 / 15					

**Table 2:** Neuropsychological and depression measures.

	**Control**		**aMCI**		**AD**				
	**Mean**	**SD**	**Mean**	**SD**	**Mean**	**SD**	**F**	**P**	**Comparisons (Bonferroni)**
N (total)	39		31		14				
MMSE (0-30)	**27.2**	2.0	**26.2**	1.9	**22.4**	3.5	22.48	*	Control ≈ MCI > AD
Digit SPAN backward	**5.8**	1.4	**4.9**	1.6	**3.7**	1.2	11.23	*	Control > MCI >AD
RAVLT (A7/A5)	**.8**	.2	**.5**	.3	**.2**	.3	26.04	*	Control > MCI >AD
Boston Naming Test	**14.4**	0.8	**13.4**	1.6	**11.6**	2.5	18.75	*	Control > MCI >AD
Phonemic verbal fluency (FAS)	**41.8**	18.3	**31.6**	15.8	**23.8**	10.9	7.25	*	Control > MCI ≈ AD
Semantic verbal fluency (animals)	**18.4**	4.4	**15.0**	4.5	**9.5**	5.5	19.71	*	Control > MCI > AD
GDS (0-15)	**4.4**	3.7	**5.3**	3.4	**3.6**	3.1	1.17	0.315	

*p value statistical significance.

### Narrative discourse assessment

All groups differed significantly for all measures of NProd and NComp. Regarding inference generation, controls performed significantly better than aMCI and AD; no differences were found between aMCI and AD subjects in this task (Table 3).

**Table 3:** Narrative discourse comparison of patients and controls.

	**Control**		**aMCI**		**AD**		***F***	***P***	**Comparisons (*Bonferroni*)**
	**Mean**	**SD**	**Mean**	**SD**	**Mean**	**SD**			
N (total	39		31		14				
Partial recount – (main ideas) (18)	**15.7**	1.7	**13**	3.7	**8.9**	3.9	28.10	*	Control > MCI >AD
Full recount of story (13)	**11.5**	1	**8.4**	3.9	**4.2**	2.9	38.97	*	Control > MCI >AD
Story comprehension (12)	**11.4**	0.7	**9.2**	2.6	**5.6**	3.4	37.02	*	Control > MCI >AD
Inference generation (total / %)	38 / 97 %		20 / 64,5 %		7 / 50 %				**Chi-square Control** > MCI ≈ AD

*p value statistical significance.

### DTI analyses

Differences in the whole brain and ROIs parameters were analyzed according to diagnostic groups.

### Whole brain analysis

**Figure 1:**
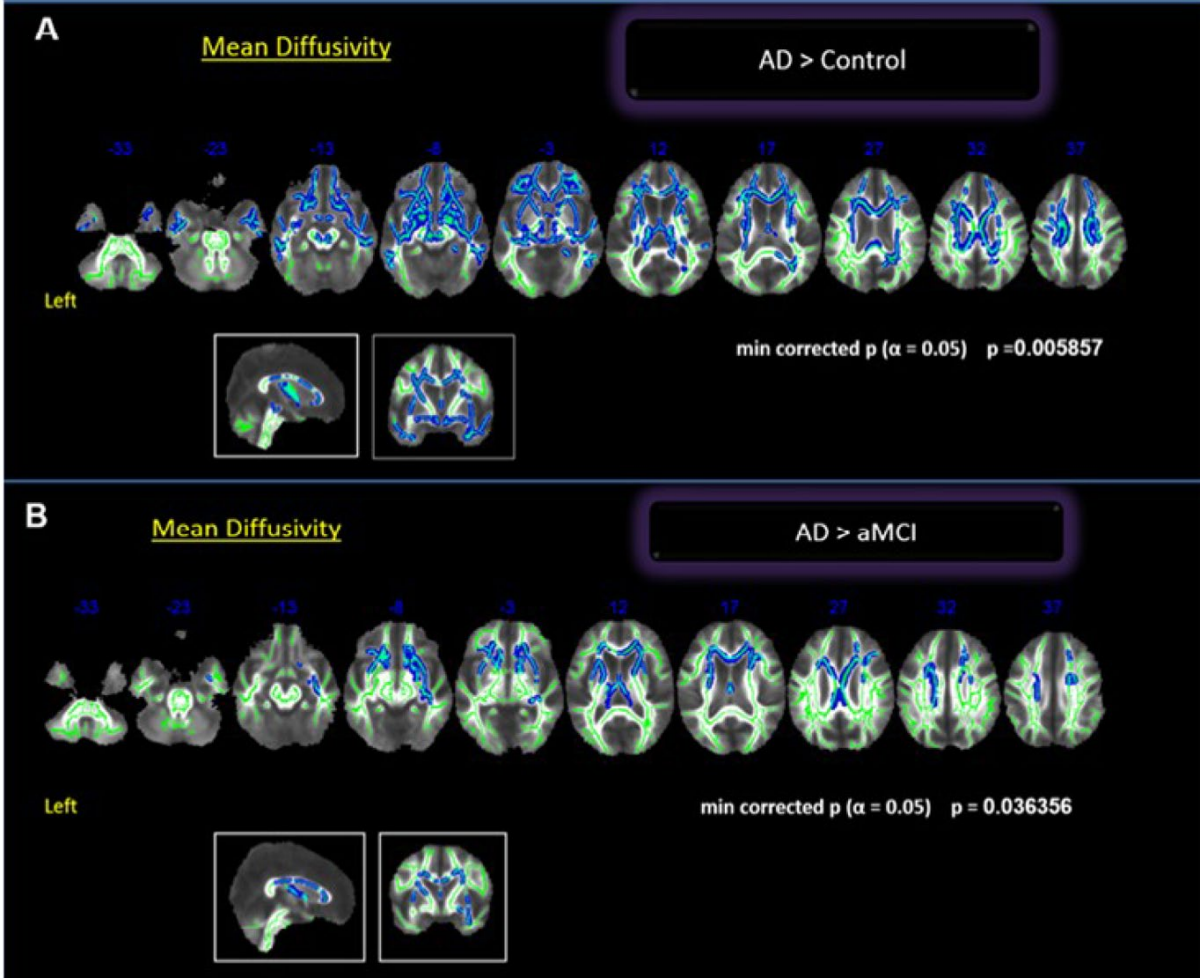
**Whole brain comparisons between groups. Cluster of voxels significantly different, corrected for multiple comparisons (*p* = 0.05) of MD values shown in blue.** (**A**) Comparison between controls and AD; (**B**) Comparison between aMCI and AD.

Comparisons between groups showed statistically significant differences between controls and AD, and between aMCI and AD, both for MD values. The AD group had globally higher MD than controls (*p* = 0.005). The difference was significant in the bilateral frontal region (orbitofrontal), bilateral temporal region (superior, middle and inferior), CC and right medial parietal lobe (precuneus and posterior cingulate) (Figure 1). We found a borderline statistical significance in the FA differences between these groups (minimum *p* = 0.054). The comparisons between aMCI and AD groups (*p* < 0.04) showed higher MD values in the AD group involving the anterior cingulate and middle frontal gyrus (Figure 1). There was no difference in the posterior lobes. We did not find differences in FA or MD values between the Control and aMCI groups.

### Regions of interest analyses

Voxelwise group comparison was performed exploring the six ROIs previously defined with statistical threshold of significance was α = 0.05. The comparison between Control and AD groups showed statistical differences in FA values in left ILF, bilateral IFOF, CC (genu and body) and right PhC. We also found statistical differences between these groups in MD values in bilateral UNC, bilateral PhC, bilateral IFOF left ILF, right SLF, and in CC (genu and body). The aMCI and AD groups showed statistical differences in FA values in bilateral SLF, right IFOF, CC (genu and body) and in MD values in all bilateral ROIs, except bilateral ILF. We found differences between Control and aMCI in FA and MD values in left ILF and left PhC.

Using a stringent cutoff (false discovery rate [FDR] q < 0.01), ROI’s voxelwise t-tests were corrected for multiple comparisons. The differences between groups were maintained between the Control and AD in three fasciculi: right UNC (*p* < 0.001); right ILF (*p* < 0.001) and bilateral PhC (right *p* < 0.001) and between aMCI and AD groups in right PhC (*p* < 0.001). There were no differences between the Control and aMCI groups.

On Supplementary Table 1, we added the results obtained in the comparison between groups for all ROI’s considering the means of FA and MD values extracted from each subject.

### Correlation between WM integrity and narrative discourse

### Whole brain analysis

**Figure 2:**
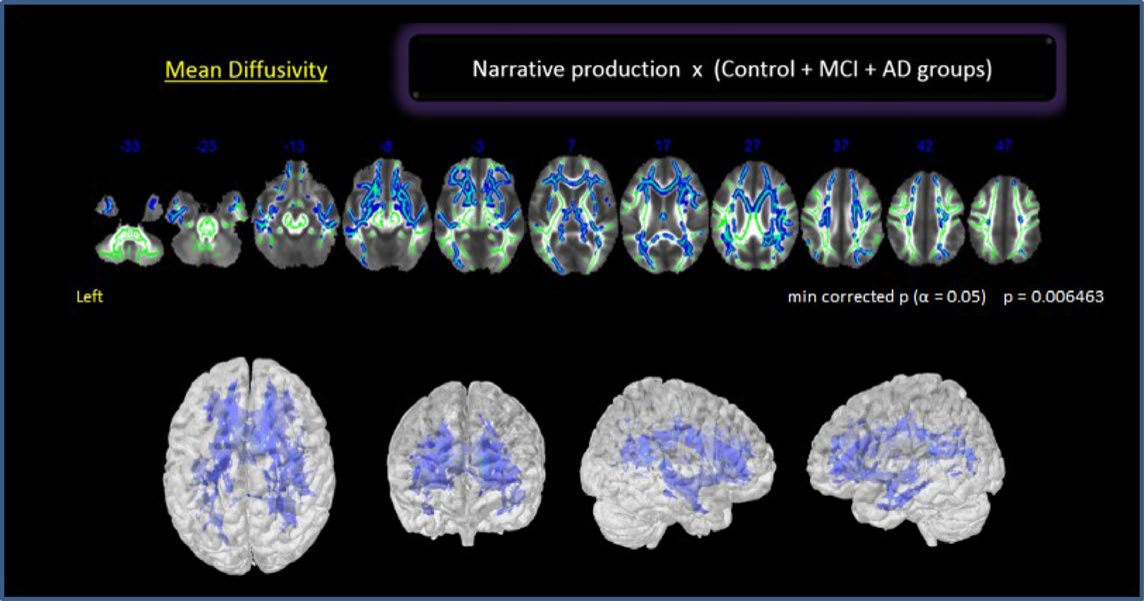
**Correlation between NProd and whole brain for all groups. Clusters of voxels significantly different MD shown in blue.**

In a preliminary analysis, we explored the whole brain with no *a priori* ROIs. There was a significant negative relationship between NProd and MD values (*p* 0.006) in bilateral frontotemporal regions and the right parieto-occipital region including the dorsal and ventral language pathways (Figure 2): left SLF (temporal, posterior division), right SLF (post central, superior parietal and angular gyrus), bilateral UNC (frontotemporal), left ILF (temporal medial region, overlapping with SLF), right ILF (temporo-parieto-occipital region up to fusiform gyrus), bilateral IFOF (orbitofrontal cortex, insula, and posterior temporal region) and genu of the corpus callosum (CC). We also found a correlation with the PhC fasciculus. No correlation was found between NComp and any specific tract.

### Regions of interest

**Figure 3:**
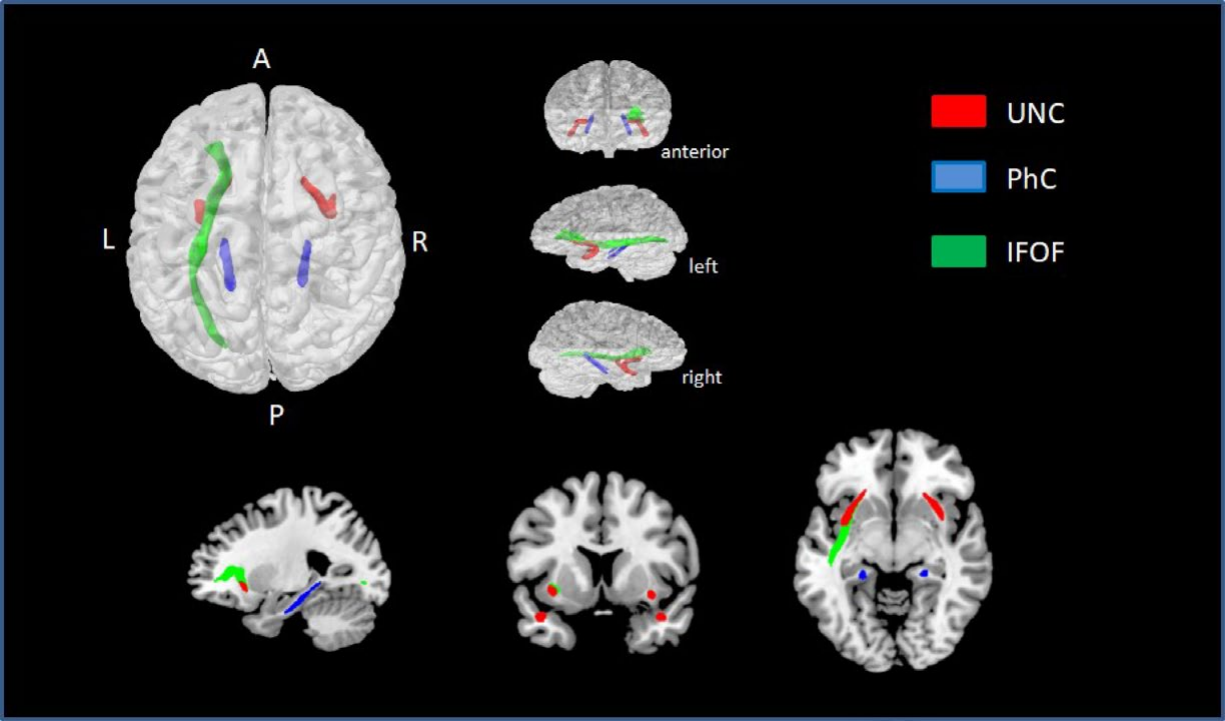
**ROIs with statistically significant correlations between MD values and NProd (FDR q < 0.01).** UNC is represented in red, PhC in blue, and left IFOF in green.

Exploring the correlation between NProd and NComp scales with different ROI’s, we found a significant correlation between NProd with FA (positive correlation) and MD (negative correlation) bilaterally in UNC and genu of CC. We also found a negative correlation between NProd and MD values in bilateral ILF, bilateral SLF, bilateral IFOF, genu and body of the CC and bilateral PhC. Significant negative correlations were found between NComp and MD values in the right UNC and left PhC. After correcting for multiple comparisons (FDR q < 0.01), we found significant correlations between NProd and MD values for UNC (left *p* = 0.003; right *p* = 0.001), PhC (left *p* = 0.000; right *p* = 0.002), and left IFOF (*p* = 0.003) (Figure 3). No correlation was found between DTI indexes and NComp in those analyses.

A linear regression for narrative discourse and all ROIs revealed a moderate negative correlation (≥ 0.3) between NProd and MD values in UNC (left *r* = 0.31; right *r* = 0.33), IFOF (left *r* = 0.30; right *r* = 0.30), PhC (left *r* = 0.37; right *r* = 0.37), and the left ILF (*r* = 0.36).

### Interference of specific cognitive domains on narrative discourse activity: dissection of the underlying neural network

Our clinical results displayed a moderate to strong significant effect on NProd by two cognitive measures: working memory (*B* = 0.357 / *adjusted R^2^ 0.117)* and verbal memory (*B = 0.550* / *adjusted R^2^ 0.302*).

Considering these results and the established importance of executive function and memory in narrative discourse capacity, we also decided to explore the influence of working memory and verbal memory in the neural correlation with narrative discourse. The results of the RAVLT (verbal memory function - retention) and Digit Span (working memory - executive function) were included as covariates in the group analysis of DTI.

**Figure 4:**
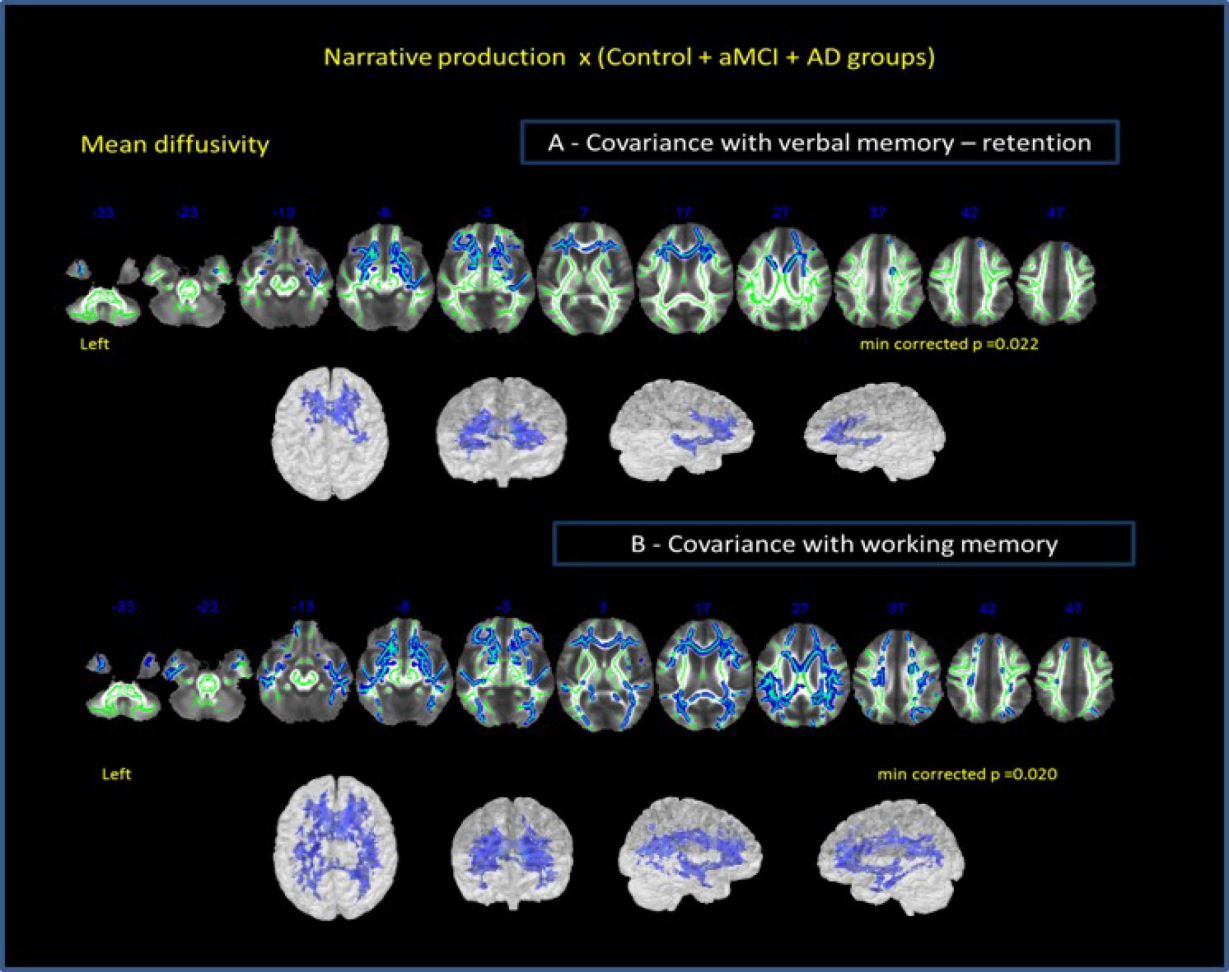
**Correlation of all groups performance in narrative discourse and whole-brain analysis.** (**A**) -verbal memory (results of RAVLT test – retention A7-A5) and (**B**) working memory (results of auditory digit span backward) as covariates. Cluster of voxels significantly different in MD value is shown in blue.

Correlation analysis among all groups performances in narrative discourse task and MD for whole brain [including verbal memory (retention A7-A5 on RAVLT) and working memory (auditory digit span backward) as covariants], indicated that verbal memory (Figure 4A) might show stronger relationship with narrative discourse than working memory (Figure 4B). NProd was also significantly associated with MD values (whole brain) (*p* = 0.02), when verbal memory was included as covariant, although restricted to anterior bilateral regions. Correlations between NProd and DTI variables (whole-brain), with working memory as covariant, showed similar negative correlation only in MD values (*p* = 0.013) and maintained almost the same results in all regions and tracts initially observed in the correlation between the NProd of all groups and DTI measures (MD values). Both domains ― narrative and working memory ― seem to be related to the same fasciculi and regions.

We also controlled the results of the association between narrative discourse and white-matter bundles for memory performance. Controlling for working memory, the results showed the same correlations between NProd and MD and FA values of ROIs observed before. In contrast, when verbal memory was considered as a covariate, we only observed correlation between NProd and MD values in anterior ROI’s. NComp was also explored with the two variables of covariance. Considering working memory as a covariate, we did not find correlation between NComp and DTI metrics of the investigated bundles. However, we found the same correlation established before amongst NComp and right UNC and left PhC (in MD values) when considering verbal memory retention as a covariate.

### CSF AD biomarkers

A subgroup of thirty-two subjects (13 controls, 11 aMCI and 8 AD) from the same previously described sample underwent CSF analysis for AD biomarkers (t-tau and Aβ1-42). Ratio t-tau/ Aβ1-42, calculated as an index of neurodegeneration, showed significant differences between Controls and AD (p = 0.03). Taken into account the white matter tracks associated with NProd, further analysis showed that CSF Ratio t-tau/ Aβ1-42 correlated with FA (UNC, IFOF and PhC) and MD values (UNC) for those tracks in ROI analyses (Supplementary Table 2).

## DISCUSSION

### Clinical findings

In this study, we investigated whether narrative discourse elicited by a heard story correlated to different AD spectrum groups (aMCI and AD).

First, we found that aMCI group presented deficits in (i) partial story recount (main ideas) (ii) full story recount (main ideas and details) and (iii) NComp, presenting an intermediary performance between controls and AD group. Moreover, aMCI group showed deficits in drawing inference in comparison to the controls, as observed in the AD group. Of note, demands involved in this kind of narrative tasks are not independent. Assuming a sociocognitive approach of discourse based on mental and situational models [42, 43], we believe, in agreement with [16], that the capacity to understand and memorize the main ideas of a heard story is the first step to a literal and non-literal understanding of the semantic meaning of the narrative such as the capacity to establish the coherent relationship between events and to draw inference. These processes consequently impact on the capacity to formulate ideas in an adequate narrative structure in different contexts (macrolinguistic aspects).

According to Ash et al. [22] the narrative deficits in AD patients as multifactorial components: episodic memory impairment, which affetcs coherence; semantic impairment, which impacts word-finding ability and limited executive function, associated with poor monitoring of the narrative. Here we propose to consider the pragmatic level of language as a fourth component. The pragmatic level could be considered a link between language and Theory of Mind. In our study, narrative discourse tasks depended on the literal, but also the non-literal and implicit content comprehension, the understanding of emotions, context, intentions and goals of the characters. The pragmatic component of discourse allows the integration of the other aspects and the generation of inference [44].

The discursive impairment of AD patients were first described in the spontaneous discourse by [45], who found deficits in local and global coherence. Since then, several other studies demonstrated discourse changes in AD using different tasks [16, 18, 21–24, 26, 46–51]. Some of the most consistent changes in early AD can be summarized as intensive repetition of information, fewer propositions, difficulties in history comprehension, in processing complex information, on establishing global coherence and cohesive links, and mental inference capacity.

Few studies showed impairments in narrative discourse capacity in aMCI subjects. Tsantalli et al. [47] used a Brief version of Boston Diagnostic Aphasia Evaluation (BDAE) to compare different language abilities in AD, aMCI and Controls. They concluded that VF, naming, writing narrative and comprehension ability associated with working memory were the most important deficits present in AD group. Oral narrative capacity was intact in aMCI; however, it is noteworthy that the narrative task was based on a description of a single visual scene. On the other hand, in the task involving semantic comprehension of the complex ideational material (comprehension of paragraphs), aMCI group presented significantly lower scores than Controls, indicating deficits in Ncomp. Both NComp and NProd were impaired in the aMCI group. Chapman et al. [16], in a study of NComp in MCI compared to AD and Controls, investigated the importance of the comprehension/retention of the gist and details in complex information processing. They found that gist information was already impaired in the MCI group with a performance similar to AD. Similarly, in our study, aMCI subjects presented difficulties in details levels, however they also showed significant impairments in main ideas. Both findings could differentiate them from Controls.

The ability to draw inference was also impaired in our aMCI group and the performance was similar to that of the AD group. In a recent study, Gaudreau [49] showed that aMCI individuals were impaired in their mental inference capacity when compared to Controls. The aMCI group presented more difficulties to identify ironic or sincere stories. This finding, as the capacity to infer the appropriate intentions from the characters of a heard story, as seen in our sample, is related to impairment in the cognitive subcomponent of the Theory of Mind (ToM). Consistently, a review conducted by Poletti et al. [52] reported several studies in which AD patients presented more deficits in this component than in the affective one. According to those authors, executive function is the most significant neuropsychological function related to ToM. However, results on MCI subjects are inconclusive regarding this aspect. Considering our sample of aMCI, we can hypothesize that several patients, besides the impairment in episodic memory, also present, to some degree, an impairment in at least one of the other multifactorial components of narrative (semantic processing, executive function or pragmatic component of language). Those narrative deficits may have determined the observed inferential difficulties and were significantly distinct from controls, not only in inference generation but also in Nprod and Ncomp.

### Classical language tasks

Although the use of classical language tests was not our objective, we compared the performance of the three groups in naming and VF considering that these two tasks, often present in neuropsychological evaluations, have been sensitive to early detection of AD [20, 47, 53, 54]. In agreement with those studies, the results from our sample showed that both naming and VF differentiate controls and AD group, but only visual naming confrontation could differentiate the three groups. There is some controversy on the sensitivity of those tests to clearly identify MCI group from normal aging subjects [24, 55–57]. Although difficulties in VF were previously described in MCI [20, 54, 58], which make it an important task to detect cognitive impairment [54], there are some differences between semantic or phonemic performances. Our findings showed that aMCI differs from controls only in phonemic VF, which could be attributed to more difficulties in executive function than in semantic associations to access and evoke words. However, those findings should be analyzed with caution, considering the wide variability of the results, as demonstrated by a large standard deviation on semantic VF performances. Accordingly, studies indicated that poor phonemic VF scores could be early identified in aMCI, indicating higher order dysfunction and risk for progression to dementia [59].

### Neuroimaging findings

### Comparison between groups

In relation to neuroimaging findings, first, we investigated the integrity of the neural language white matter network in aMCI and AD patients applying the WB and ROI’s DTI analyses.

Our findings on WB analysis showed that the Control and AD groups differed in WM measures, which is in agreement with several others studies showing decreased FA/Increased MD in AD patients in bilateral frontotemporal e parietal regions [31, 33, 60–64]; also, they seem to be more pronounced in right regions [65]. In our sample, the main differences occurred in MD measures. We had a borderline statistical result in those bilateral regions (p= 0.054) considering FA measures. Moreover, compared to aMCI, AD subjects showed increased MD in the language white matter tracks. The aMCI and AD groups did not differ in posterior regions, suggesting that the differences between Controls x aMCI and AD were more evident in the anterior regions (anterior cingulate and middle frontal gyrus). Following this reasoning, it was expected to find some differences between control and aMCI in the posterior regions, but it did not reach significance in WB analysis, only in ROI’s analysis.

Exploring all six previously specified tracks defined as part of the language network, ROIs analysis showed differences in both FA and MD values, when AD subjects were compared to controls. These differences occurred in left ILF, bilateral IFOF, CC (genu) and right PhC in agreement to other studies [31, 66]. The aMCI group also differed from controls and AD subjects. Compared to controls, aMCI patients showed increased MD and decreased FA in ILF and PhC, indicating the expected differences between these groups in posterior regions. While the PhC is a short and inferior cingulate bundle specifically related to the medial temporal region, the ILF is a visual association pathway with long fibers that connect, by ventral pathway, the occipital and temporal regions, being one of the main components of WM in this posterior region [34]. The differences between aMCI and AD were present in FA and MD measures in all ROI’s, except in bilateral ILF. Those results confirmed the greater WM abnormality presented by clinical groups in posterior regions than anterior regions when compared to controls, in agreement to consistent findings concerning the WM changes in MCI and AD individuals [67, 68]. However, the specific location of the WM abnormalities in aMCI individuals are not clearly established. Zhuang et al. [62] cited several studies in which inconsistent results related to the WM integrity of MCI were presented, possibly associated to the heterogeneity of MCI groups. Another reason would be the different ROI’s studied and small sample sizes [63].

### Correlation with narrative discourse

The correlation between narrative discourse and WM integrity across all groups was seen in our study both in WB and ROI’s analysis. Although the biological basis of the DTI derived metrics is not specific and can be sensitive to tissue specificity and quality of data acquisition and processing, alteration of WM integrity has been correlated to changes in FA and MD values [69]. However, while there are few studies correlating specific language deficits and WM diffusion in different neurological disorders, such as aphasia [70, 71] and autism [72, 73], to the best of our knowledge this is the first study investigating the relationship between narrative discourse and WM integrity in aMCI and AD groups.

The correlations found between NProd and WM integrity in WB assessment in following bilateral regions were expected, considering the dimensional analysis of the sample and the multifactorial components associated in discourse performance: the left fronto-temporal regions directly involved in language, memory and executive processes and the right hemisphere involved in discursive practices. Some studies using fMRI have shown that NComp recruits the bilateral superior temporal areas implicated in word and sentence comprehension [74], but also other cortical regions such as prefrontal cortex, anterior temporal lobe, temporo-parietal junction and cingulated areas [44, 75, 76]. Involvement of those areas on the left and/or right hemispheres depends on the type and the complexity of the task. The more complex the demand on narrative discourse, the greater the participation of the right hemisphere [29]. When a task demands inferences drawing (e.g., of a third party’s intentions or feelings), similarly to our task, there is recruitment of prefrontal cortex, right temporo-parietal junction and anterior cingulate [44, 76]. A fronto-parietal network is related to inferential process and global coherence [75]. The posterior cingulate cortex, which is associated with different cognitive domains such as memory, language, executive function and visuospatial perception [77], is recruited by both comprehension and production of narrative. It has been also implicated in memory processing during communication [78] and global coherence of narrative [79, 80]. The severity of the deficits in comprehension and narrative production in AD probably increases according to the progression of damage in cerebral cortex and related subcortical structures [81]. Findings based on fMRI studies suggested the existence of a specific fronto-temporal-parietal network underpinning discourse processing which is represented in both cerebral hemispheres.

ROI’s analysis allowed the distinction of more specific networks involved in NProd than in NComp. After a stringent correction for multiple comparisons, the correlation with NProd persisted in three fasciculi: bilateral UNC, bilateral PhC and left IFOF. The UNC integrates a ventral pathway and connects the anterior temporal lobe to the frontal lobe. It has been described as supportive of “sound to meaning maps” [82] and it is associated with sentence comprehension, linking the syntactic to the semantic domains [37, 83]; semantic processing [37] and episodic memory [31]. The left IFOF is a long fiber connecting the occipito-temporal regions to anterior temporal until the orbito-frontal cortex and also participates in the semantic network of semantic memory [84] and visual and auditory associations [34]. The two pathways (IFOF and UNC), are associated with semantic processing and episodic memory which are important subcomponents of narrative discourse. The PhC, a division of the cingulate bundle, is part of an important network connecting the temporal, parietal and frontal regions [81, 85]. Its functional connectivity differs according to the involvement of the anterior and posterior regions, and the visual, spatial and contextual associations. The importance for scene understanding and recognition depends on the interaction among different cortical networks [86]. In order to provide an adequate NProd, the individual needs to establish a mental representation involving the association of a large number of contextual information and for this, the declarative memory – both episodic and semantic – must be recruited. The mesial temporal lobe comprising the entorrinal, perirrinhal and parahipocampal subregions, along with the hippocampus, plays an important role in declarative memory [87]. The participation of bilateral parahippocampal area in the contextual associative memory was demonstrated in a recent fMRI study [88]. Therefore, the bilateral pathways comprising UNC and PhC seem to be the WM network involved in narrative discourse elicited by a heard story. The left hemisphere is involved in literal comprehension and other cognitive processes, such as memory, whereas the right hemisphere is more implicated with inference generation and non-literal meaning [75]. The bilateral frontal regions, connected to posterior regions by UNC and IFOF, are involved in semantic-pragmatic and executive function components of NProd.

Our additional covariance analysis demonstrated that episodic and working memories (Figure 3) contributed differently to the correlation between WM and narrative discourse. As expected, the correlation disappeared in posterior regions where we could find a structural cortical and WM association with episodic memory [87, 89]. We did not find significant differences in the correlations using working memory as a covariate. Previous studies have shown that working memory is an important predictor of narrative comprehension [75]. Working memory and narrative discourse are parts of the same construct – the executive function [90] and they share the same neural substrates: frontotemporal and parietal regions [75, 90, 91]. Differently from working memory, episodic memory demonstrated independent correlations with the pathways associated with narrative.

Finally, CSF AD biomarkers (t-tau/Aβ1-42 index) were significantly associated with DTI measures (FA and MD) in language-related tracts (UNC, IFOF and PhC). This finding suggested a relationship between WM damage and primary neuronal loss in subjects with different levels of cognitive impairment within the AD spectrum. Our findings are in line with previous reports, which indicated associations among CSF AD biomarkers (t-tau, phosphorylated –tau; Aβ-42 and t-tau/ Aβ-42 ratio) and decreased FA or increased MD in specific ROI’s bundles, such as the cingulum [92], the parahippocampal gyrus, the precuneus and the inferior temporal regions [93].

This study had some limitations. First, this is a cross-sectional study with a somewhat small sample. For this reason, we could not investigate different clusters within the aMCI group (e.g., single or multiple affected domains). Second, since inference generation is measured as a dichotomous variable, correlation analysis between this parameter and the DTI measures could not be performed. New studies using a larger number of patients will be important to support our initial results. Finally, data on CSF AD biomarkers were only available for a subset of subjects in our sample, which might limit the validity of those results. Those shortcomings should be considered for future studies in the field.

To conclude, our findings indicate that aMCI individuals, similarly to what has been observed in those with AD, portray difficulties in narrative discourse elicited by a heard story involving oral comprehension, production and ability to inference generation. Such ecological language assessment could contribute to early identification of cognitive decline. The intermediary performance presented by aMCI when compared to controls and AD groups reveals a dimensional characteristic of the narrative discourse impairment in this neurodegenerative disease. These deficits could be correlated to the abnormalities observed in white matter integrity in ventral pathways for language processing (IFOF and UNC) and inferior segment of cingulum (parahipocampal) in bilateral frontotemporal and parietal regions. In conclusion, our study indicated that the severity of deficits in narrative discourse was mirrored by neuroimaging and CSF biomarkers changes in the AD continuum.

## MATERIALS AND METHODS

### Participants

From a total sample of 225 individuals initially enrolled for a research project on aging and cognition, developed at the D’Or Institute of Research and Education (IDOR) in Rio de Janeiro, Brazil, eighty-four participants were included. Almost 10% of the sample (20 individuals) gave up before completing the defined time length research protocol and 88 participants were excluded according to the criteria specified below.

Subjects were volunteers referred to the service by physicians or other health professionals. All individuals underwent psychiatric and neurological evaluation, followed by neuropsychological and language assessments. The latest included a narrative discourse task which was videotaped and rated by an experienced speech-language therapist according to objective criteria. All participants were assessed for vision and hearing disabilities.

Subjects were categorized as healthy control, aMCI or AD in a weekly based meeting coordinated by a senior-certified psychiatrist (P.M.). The Winblad et al. criteria [94] were adopted for the diagnosis of aMCI. Memory impairment was objectively defined as performance below 1.5 SD for age on the delayed recall Logical Memory and Visual Reproduction subtests of the Wechsler Memory Scale (WMS-III). AD was diagnosed according to criteria included in the fifth edition of the Diagnostic and Statistical Manual of Mental Disorders (DSM-5) for probable major neurocognitive disorder due to AD [95].

**Figure 5:**
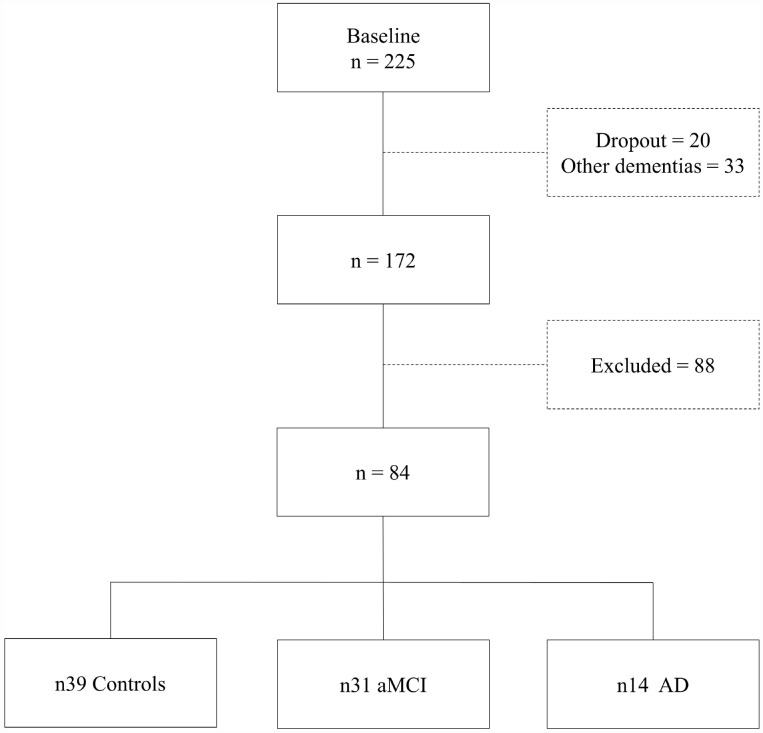
**Sample selection diagram.**

The eighty-four volunteers (39 controls, 31 aMCI and 14 AD – (Figure 5) were matched for age, sex and education. They qualified for the study according to inclusion criteria: native Brazilian Portuguese as their first language; aged between 60 and 85 years, formal education equal to or greater than eight years; completed language and neuropsychological batteries and MRI evaluation. Exclusion criteria were Clinical Dementia Rating (CDR) scores higher than 1, frontotemporal dementia, primary progressive aphasia, dementia with Lewy bodies, advanced cerebrovascular disease, non-amnestic MCI and any major neuropsychiatric disorder report (schizophrenia or other psychotic illness, bipolar disorder, epilepsy, alcohol and drug abuse, current severe depressive disorder and severe head injury).

All participants provided written informed consent before entering the study, which was previously approved by the IDOR Research Ethics Committee (CEP 226/11).

### Cognitive and behavioral assessments

Narrative discourse was evaluated with the story “Mark and his wheel” Brazilian population (MAC Battery - Montreal Communication Evaluation Battery – [95, 96]). This story evaluates the NProd and Ncomp elicited by auditory-verbal stimuli. The story has five paragraphs following a chronological sequence. The task had four subsequent steps:

a) Partial Recount: the story was told by paragraphs. The participant was asked to recount the paragraph in his own words immediately at the end of each paragraph. We recorded all the information – main ideas and details (maximum 29) and the total of essential information (main ideas) of the story (maximum 23).

b) Full Recount: After the recount by paragraphs, the participant was asked to hear again the complete story without breaks. At the end, he was asked to recount the full story with his own words. We registered the total information reported based on 13 previously determined units considered necessary for adequate recount of the story.

c) NComp: The participants answered 12 predefined questions of narrative understanding. Correct, incorrect and missing answers were registered.

d) Inference: After the full recount of the story, the participant was asked to provide a title for the story. The ability to draw a correct inference (classified as “correct” or “incorrect”) was based on two out of three objective indexes: overt expression during the task (laugh, facial expression or comment denoting the moment of the experience of insight into the gist of the story), title given to the story, and response to the last two questions (item c, above).

The researcher who conducted the experiment was trained to administer, analyze and score the assessment protocol; evaluations of the first six patients were checked by the author of the standardized test who endorsed the researcher’s analyses.

Boston Naming Test (short form) [97] was used to evaluate visual confrontation naming. The unconstrained verbal fluency (VF) [96], semantic VF (animals) and phonemic VF(FAS) [98] were used to evaluate lexical retrieval. Neuropsychological assessment included a test of verbal memory – Rey Auditory Verbal Learning Test (RAVLT) [99] and verbal working memory (digit span backward). General cognitive screening was evaluated through the Mini-Mental State Examination (MMSE) [100]. Depressive symptoms were investigated with the Geriatric Depression Scale (GDS) [101]. As previously cited, participants were staged for the presence of cognitive impairment and dementia using the CDR.

### Neuroimaging acquisition and processing

### Data acquisition

All subjects underwent imaging acquisition protocol in a 3 Tesla magnetic resonance scanner (Achieva, Philips Medical Systems) including isotropic high-resolution 3D T1-weighted sequence (TR/TE 13/ 1.4 ms; matrix 256 x 256 mm; FOV 240 mm; slice thickness 1 mm; 140 slices) and a DTI sequence (axial single-shot, spin-echo, echoplanar sequence; isotropic voxel size of 2.5 mm, 60 slices, repetition time (TR)/echo time (TE): 5582/65 ms; field of view (FOV) 240 mm; matrix 96 x 96 mm. Diffusion sensitization gradients were applied in 32 non-collinear directions, with a factor of 1000 s/mm².

### DTI processing and analyses

Prior to analysis, patients and control datasets were anonymized. To ensure for quality of acquired data, for each subject, and before estimating the specific diffusion maps, all diffusion images were visually inspected for artifacts detection. DTI data processing was carried out using FMRIB’s Diffusion Toolkit (FDT), part of FMRIB software Library (FSL, RRID:SCR_002823; http://www.fmrib.ox.ac.uk/fsl/) version 5.0, using standard well established protocols, including all steps related to quality control in post-processing analyses. Non-diffusion and diffusion images were co-registered to correct for movement artifacts and eddy current distortion effects on EPI readout. The six independent elements (three eigenvectors - v_1_, v_2_, v_3_ - and three eigenvalues - λ_1_, λ_2_, λ_3_) of the diffusion tensor were calculated from each diffusion-weighted image after removing non-brain tissue with Brain Extraction Tool (BET - Brain Extraction Tool, RRID:SCR_014586; http://poc.vl-e.nl/distribution/manual/fsl-3.2/bet2/) as a part of FSL software. After the fractional anisotropy (FA) and mean diffusivity (MD) maps were calculated from the eigenvalues, color-coded maps were generated from the FA values and three vector elements of v_1_ to visualize the WM tract orientation.

To preserve the original white matter structure, keeping the overall tracts intact as much as possible, a voxelwise specifically tuned nonlinear registration method was used to align FA images of all subjects into a standard space. For each statistical analysis, FA data of each subject were aligned to every other one and the most representative was used as the target image. This target image was affine-aligned into standard space and every image was transformed into a 1mm x 1mm x 1mm standard space by combining the nonlinear registration to the target FA image with affine transform from target to standard space. All the registration steps were submitted to a batch system for parallel processing. The upsampled FA maps were averaged to create the mean FA from all subjects. The mean FA was then used to generate the ‘‘skeleton tract’’, which represents the tracts shared by all subjects (mean FA thresholded to 0.2). Finally, registered FA data from each subject were ‘‘projected’’ onto the mean FA skeleton mask to generate the final skeletonized FA data. The nonlinear warps and skeleton projection obtained from FA images transformations were also applied to individual MD maps.

Usually, even indirectly, values of DTI metrics are correlated to the brain microstructure tissue organization. And, although not specific, usually increased MD and decreased FA in WM tracts can be associated to changes in the tract organization, such as less myelination and /or decreased axonal integrity [69]. To explore the WM integrity and differences among AD, aMCI and controls, we analyzed FA and MD WM tracks values, based on ROI analysis that were chosen according to their relationship with language performance: UNC, SLF (arcuate), ILF, IFOF, CC, and the parahippocampal division of the cingulate bundle [102], from now on called “parahippocampal cingulate” (PhC) using the JHU DTI-based WM atlases, included in FSL database (https://www.hopkinsmedicine.org).

In addition, to examine the relationships between WM integrity and narrative discourse, analyzed correlations between narrative measurements and all groups FA or MD values calculated from the same ROIs. These analyses were also performed taking to account the verbal memory, working memory and executive function confounding effect (applying regression analysis).

To compare groups’ differences in the 6 fasciculi previously defined and to investigate the relationship between tracts and narrative performance we applied a statistical significance level of α = 0.05, corrected for multiple comparisons analyses.

To test for significant FA and MD global differences among AD, aMCI and control, a voxelwise cross-subject statistical analysis was also carried out using permutation-based non-parametric inference with 5,000 random permutations (FSL Randomise tool) on each voxel of the resulting ‘‘mean FA skeleton’’ mask of the whole brain. The results were considered significant at *p* < 0.05, using Threshold-Free Cluster Enhancement (TFCE) fully corrected for multiple comparisons (Family-wise Error Rate, FWE). The thresholded-skeletonized resulting image was thickened for better visualization. Further we have looked for correlations between narrative and all groups FA or MD values at each voxel of the mean-FA skeleton mask (whole-brain analysis).

### CSF AD biomarkers

CSF samples (15 ml) were collected through lumbar puncture at the L3–4 or L4–5 interspace by a trained neurologist and were immediately stored at 4°C. Within 2 hours collected CSF was centrifuged at 2,000 g for 10 minutes at room temperature. Samples were aliquoted in 0.5 ml aliquots and stored immediately at -80°C till use.

All lumbar punctures were performed around 11 a.m. to minimize possible circadian fluctuations in the biomarker levels. Aβ1-42 and t-tau concentrations were measured using Euroimmun enzyme immunoassays with single antigen (ELISA) kits.

### Statistical analyses

Data analysis were performed using the Statistical Package for the Social Sciences (SPSS, RRID:SCR_002865; http://www-01.ibm.com/software/ uk/analytics/spss/, https://www.ibm.com) software, version 21.0. ANOVA with post-hoc Bonferroni tests were used for all parametric variables’ analyses. For the categorical variables (inference generation), we used Pearson’s Chi-square test. Comparisons between groups and correlations between narrative discourse and white matter integrity considering FA and MD values were performed for the entire sample and for each group in separate. Linear regression was performed to verify the correlation between performance in narrative discourse and other cognitive functions, such as memory and executive functions. Stepwise regression analysis of verbal memory and working memory were performed to define the main predictors to the narrative discourse (dependent variable). For all statistical tests, we adopted a level of significance (α) of 0.05, with corrections.

## Supplementary Material

Supplementary Tables
