# Supplementary material for: Narrative impairment, white matter damage and CSF biomarkers in the Alzheimer’s disease spectrum
Source: Aging (Albany NY). 2019 Oct 31;11(20):9188–208. doi: 10.18632/aging.102391 (PMC6834410; doi:10.18632/aging.102391)
Supplement: Supplementary Tables [file aging-11-102391-s001.pdf]

SUPPLEMENTARY TABLES

Supplementary Table 1. Summary of comparison between all groups in different ROI's: mean and standard error in FA and MD.

ROIs		Controls		aMCI		DA		Comparison p< 0.05
		FA	MD	FA	MD	FA	MD	
		Mean (SE)						FA * MD **
UNC	L	0.350 (±0.006)	0.850 (±0.009)	0.346 (±0.006)	0.858 (±0.009)	0.333 (±0.008)	0.895 (±0.020)	Control < AD**
	R	0.349 (±0.005)	0.824 (±0.008)	0.344 (±0.006)	0.845 (±0.009)	0.322 (±0.010)	0.901 (±0.025)	Control < AD** aMCI< AD **
SLF	L	0.386 (±0.004)	0.761 (±0.006)	0.383 (±0.005)	0.757 (±0.010)	0.362 (±0.10)	0.801 (±0.018)	Control < AD** aMCI< AD **
	R	0.403 (±0.005)	0.738 (±0.005)	0.404 (±0.007)	0.748 (±0.010)	0.374 (±0.014)	0.805 (±0.030)	Control < AD** aMCI< AD **
ILF	L	0.324 (±0.003)	0.780 (±0.005)	0.316 (±0.004)	0.796 (±0.006)	0.303 (±0.008)	0.829 (±0.030)	Control > AD* Control < AD** aMCI < AD**
	R	0.360 (±0.003)	0.753 (±0.005)	0.360 (±0.004)	0.767 (±0.007)	0.351 (±0.08)	0.785 (±0.014)	Control < AD**
IFOF	L	0.360 (±0.003)	0.798 (±0.005)	0.354 (±0.005)	0.812 (±0.005)	0.344 (±0.008)	0.843 (±0.018)	Control < AD**
	R	0.355 (±0.004)	0.807 (±0.006)	0.360 (±0.005)	0.811 (±0.008)	0.340 (±0.010)	0.867 (±0.025)	Control < AD** aMCI < AD**
CC	genu	0.409 (±0.005)	0.970 (±0.010)	0.411 (±0.006)	0.958 (±0.013)	0.381 (±0.010)	1.025 (±0.024)	Control > AD* Control < AD**
	body	0.435 (±0.006)	1.096 (±0.014)	0.450 (±0.007)	1.103 (±0.015)	0.415 (±0.128)	1.180 (±0.028)	Control > AD* Control < AD** aMCI < AD**
	splenium	0.436 (±0.005)	0.920 (±0.012)	0.444 (±0.006)	0.925 (±0.016)	0.416 (±0.015)	0.970 (±0.034)	
PhC	L	0.272 (±0.005)	0.979 (±0.012)	0.266 (±0.005)	0.820 (±0.011)	0.256 (±0.010)	0.891 (±0.027)	Control < AD** aMCI < AD**
	R	0.303 (±0.006)	0.827 (±0.010)	0.303 (±0.006)	0.843 (±0.017)	0.270 (±0.010)	0.994 (±0.043)	Control > AD* aMCI > AD* Control < AD** aMCI < AD**

P-value <0.05 / MD values (10^{-3} mm/s).
*p value statistical significance.
Abbreviations: aMCI: Amnestic Mild Cognitive Impairment; AD: Alzheimer's Disease; CC: corpus callosum; CDR: Clinical Dementia Rating; CSF: cerebrospinal fluid; DTI: Diffusion Tensor Imaging; FA: fractional anisotropy; fMRI: functional magnetic resonance imaging; GDS: Geriatric Depression Scale; IDOR: D'Or Institute of Research and Education; IFOF: inferior fronto occipital fasciculus; ILF: inferior longitudinal fasciculus; MCI: Mild Cognitive Impairment; MD: mean diffusivity; MMSE: Mini-Mental State Examination; NComp: narrative comprehension; NProd: narrative production; PhC: parahipocampal cingulate; RAVLT: Rey Auditory Verbal Learning Test; ROI: regions of interest; SLF: superior longitudinal fasciculus; ToM: Theory of Mind; UNC: uncinate fasciculus; VF: verbal fluency; WB: Whole brain; WM: White Matter.

Supplementary Table 2. Association between ROIs and Ratio t-tau/ Aβ1-42.

Correlation T-tau /Aβ1-42 x ROI's		Min corrected p (α =0,05)	
**ROI's**	**Correlation**	**FA**	**MD**
UNC	positive	0,62452	**0,001212***
(R)	negative	**0,000806***	0,95617
UNC	positive	0,904262	0,110281
(L)	negative	0,076752	0,977378
IFOF	positive	0,757019	0,066855
(R)	negative	**0,026863***	0,767724
IFOF	positive	0,935569	0,075742
(L)	negative	0,089073	0,997172
PhC	positive	0,719451	0,09998
(R)	negative	0,533428	0,864472
PhC	positive	0,931529	0,175318
(L)	negative	**0,036962***	0,932337

*p value statistical significance.
